# Rooks in Brighton

**Published:** 1919-04-26

**Authors:** 


### ROOKS IN BRIGHTON.

It has been the writer's privilege to have a small rookery in a public garden of Brighton under daily observation for a year. The rookery has had its present position for just over a century. The fact is historical and is recorded in the archives of the town.

The 18th of February 1918 was the first day on which he walked beneath the trees. Some rooks were building busily, but all that eventually had their habitations there had not begun their nests. It seemed that the early builders were more leisurely at their work than some that started operations later, but so far as stability was concerned there was no noticeable difference between nests more hastily run up and those finished with deliberation. The sticks of the nests were broken from trees growing in the gardens. Those twigs that fell plainly showed the marks of the strong beaks that had partly cut them through and partly broken them off. It was curious to notice that sticks that were dropped were never gathered up from where they fell, nor did it seem that any sticks were picked up from off the ground. All used were twigs fresh taken from the trees. Much of the lining material was gathered from the lawns, especially from spaces beneath bushes where the grass had withered in the winter. The stuff taken was dry, dead, fine, fibrous grass. It was collected in big beakfuls and carried on frequent journeys to the nest.

There were birds brooding at the end of March, but even then all the nests were not finished. In accordance with the difference of dates of laying, there was a considerable difference between the date of the appearance of the first young rook and the last.

As soon as the "branchers" had got strength for a journey they and their parents went off beyond the borders of the town and did not return, so it came about that the colony got less and less, and by mid-June the rookery was deserted. No rook visited it again till November, but in that month on fine days a few would come usually of an afternoon. It was noticeable that if the day was fine and gave at all that pleasant feeling called "springlike," it was certain that some four or five rooks would visit some of the nests. But if the day were wet or cold none would appear. In December the visits seemed more frequent and came to be on worse days than in the month before, but still no birds came on a really bad day. When the birds came, one or two would sit each on a nest, whilst others perched about near on the branches. That on the nest would caw and spread its tail and elevate it and depress it vertically with somewhat jerky movements and bow as it cawed. Those around sometimes made reply, but were less vociferous. The idea of conversation was conveyed by these actions. No work of any kind seemed to be done during these visits till January 17 this year. On that day some of the rooks definitely begun work on some of the old nests, throwing down from them a good many rotten twigs and dusty material that may have been the rotten linings. But the work done was very slight and only continued for a short hour each day. Then came a snap of cold and some snow on which the work and the visits ceased. For a week or more before February 6, the birds, as if daunted by the intemperate season, have stayed away altogether, but doubtless, should the wind bear to the west and the sun shine out again, they will return and set to nesting in earnest.

